# LPA Is a Chemorepellent for B16 Melanoma Cells: Action through the cAMP-Elevating LPA_5_ Receptor

**DOI:** 10.1371/journal.pone.0029260

**Published:** 2011-12-14

**Authors:** Maikel Jongsma, Elisa Matas-Rico, Adrian Rzadkowski, Kees Jalink, Wouter H. Moolenaar

**Affiliations:** Division of Cell Biology, The Netherlands Cancer Institute, Amsterdam, The Netherlands; University of Oldenburg, Germany

## Abstract

Lysophosphatidic acid (LPA), a lipid mediator enriched in serum, stimulates cell migration, proliferation and other functions in many cell types. LPA acts on six known G protein-coupled receptors, termed LPA_1–6_, showing both overlapping and distinct signaling properties. Here we show that, unexpectedly, LPA and serum almost completely inhibit the transwell migration of B16 melanoma cells, with alkyl-LPA(18∶1) being 10-fold more potent than acyl-LPA(18∶1). The anti-migratory response to LPA is highly polarized and dependent on protein kinase A (PKA) but not Rho kinase activity; it is associated with a rapid increase in intracellular cAMP levels and PIP3 depletion from the plasma membrane. B16 cells express LPA_2_, LPA_5_ and LPA_6_ receptors. We show that LPA-induced chemorepulsion is mediated specifically by the alkyl-LPA-preferring LPA_5_ receptor (GPR92), which raises intracellular cAMP via a noncanonical pathway. Our results define LPA_5_ as an anti-migratory receptor and they implicate the cAMP-PKA pathway, along with reduced PIP3 signaling, as an effector of chemorepulsion in B16 melanoma cells.

## Introduction

Lysophosphatidic acid (LPA) is a multifunctional lipid mediator that stimulates migration, proliferation, survival and other functions in many different cell types, both normal and malignant. LPA acts through six known G protein-coupled receptors (GPCRs), termed LPA_1–6_, which show both overlapping and distinct signaling properties and tissue distributions [1], [2]. The three classical and best studied LPA receptors, LPA_1–3_, belong to the so-called Edg subfamily of GPCRs. Three additional LPA receptors, termed LPA_4_ (formerly P2Y9), LPA_5_ (GPR92) and LPA_6_ (P2Y5) stand apart from the Edg family and are more closely related to the purinergic receptor family, strongly suggesting that LPA receptors have evolved from distinct ancestor genes [1], [2]. LPA receptors couple to multiple G protein-effector pathways, accounting for the multitude of cellular responses to LPA.

LPA is a major constituent of serum [3] and is produced through the hydrolysis of lysophosphatidylcholine (LPC) by a secreted lysophospholipase D, named autotaxin (ATX), originally identified as a motility-enhancing factor for melanoma cells (reviewed in [4], [5]). The ATX-LPA signaling axis is of vital importance for embryonic development [4] and is an effector of tumor growth, angiogenesis and metastasis in mice [6]–[11].

Cell migration plays a key role in embryogenesis, tissue renewal, immune responses and cancer metastasis. Most tumor cells, including carcinoma, lymphoma, glioma and melanoma cells, show an enhanced migratory response to LPA (positive chemotaxis) [12]–[17]. Where examined, LPA-induced migration is primarily mediated by Edg-family LPA_1_ and LPA_2_ receptors and involves both G_i_- and G_12/13_-mediated signaling pathways. One non-Edg-family receptor, notably LPA_4_, has been reported to suppress cell migration [18]. In general, cell migration is driven by pathways controlled by Rho GTPases and phosphatidylinositol 3-kinase (PI3K), acting in a coordinate fashion to control the spatiotemporal dynamics of cytoskeletal components. However, soluble second messengers such as Ca^2+^ [19], [20] and cyclic AMP (cAMP) [21], [22] also play crucial roles in directing cell migration and chemotaxis.

Here we show that, unexpectedly, LPA strongly impedes the basal and growth factor-induced migration of B16F10 melanoma cells. We show that the inhibitory effect of LPA is mediated by the LPA_5_ receptor and that a rise in cAMP with consequent activation of protein kinase A (PKA) is an important effector of LPA_5_-mediated chemorepulsion, with a possible additional role for reduced phosphatidylinositol (3,4,5)-trisphosphate (PIP3) signaling. Our results identify LPA_5_ as an anti-migratory receptor and they point to a mechanism of LPA-induced chemorepulsion likely to be relevant for tumor cells that predominantly express LPA_5_, acting to override positive chemotactic signals.

## Results and Discussion

### LPA, serum and autotaxin inhibit B16 cell migration in a highly polarized fashion

**Figure 1:**
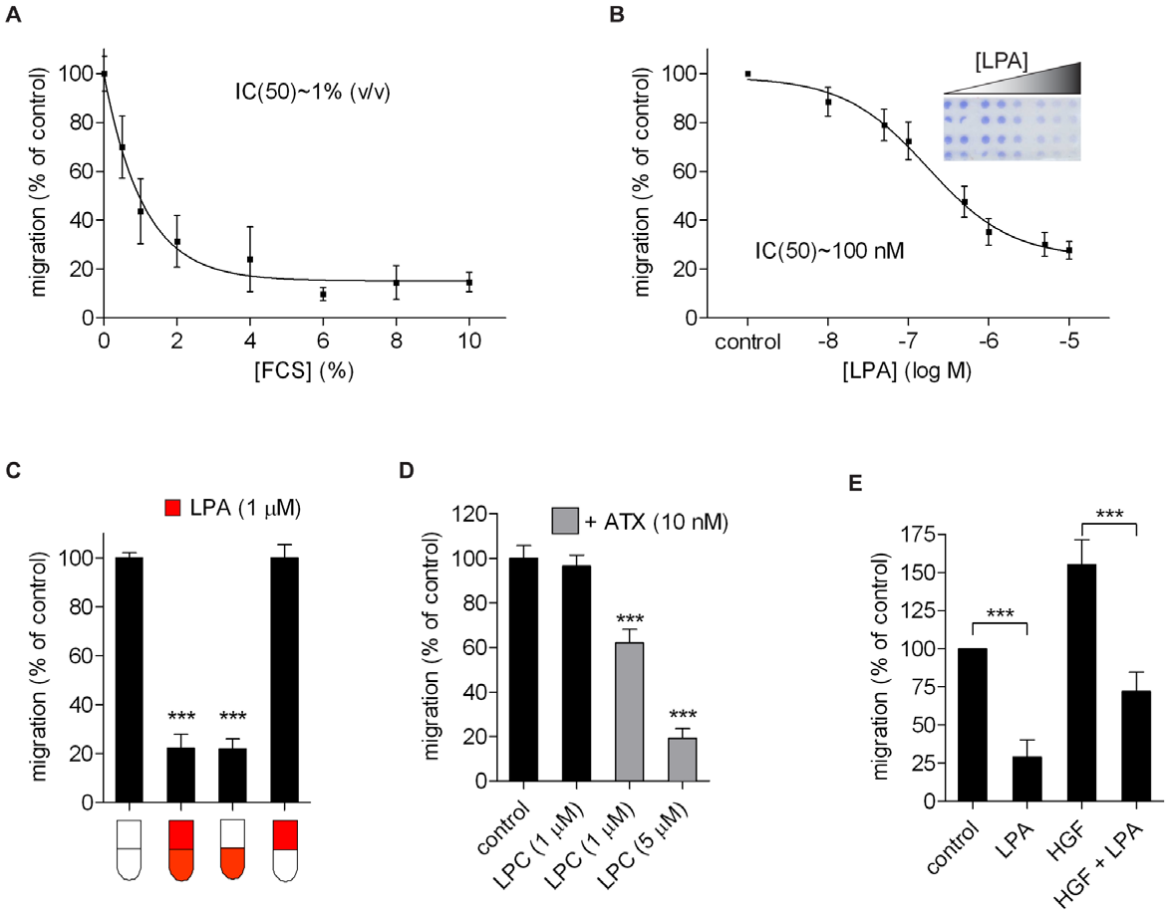
LPA-induced inhibition of B16F10 cell migration. B16F10 cells were allowed to migrate for 3 hrs through fibronectin-coated 8 µm porous membranes. **A–B.** Inhibition of cell migration by increasing concentrations of fetal calf serum (FCS) (**A**) or 1-oleoyl-LPA (**B**). The inset in **B** shows a representative transwell filter after staining. **C.** Cell migration was assayed using LPA (1 µM) in either the upper or lower chamber, or in both chambers, as schematically indicated by the red color. **D.** B16F10 cell migration in the presence of LPC (1 or 5 µM) with or without added recombinant autotaxin (ATX; 10 nM) in the lower chamber. **E.** Enhanced cell migration (chemotaxis) induced by HGF (50 ng/ml) and inhibitory effect of LPA (1 µM). Values are the means ± SEM of at least three independent experiments and were normalized to the migration of non-stimulated cells (*** P<0.0001).

When testing various tumor cell types for their chemotactic response to 1-oleoyl-LPA (LPA(18∶1)) and serum (FCS) in transwell assays, we found that B16F10 melanoma cell migration is strongly inhibited by both FCS and LPA(18∶1) (Figure 1A,B). Under serum-free conditions, B16 cells show a high rate of basal, ‘spontaneous’ transwell migration during a 3 hr assay period. Addition of FCS or LPA to the lower transwell chamber inhibited the basal migration of B16 cells almost completely. Half-maximal inhibitory effects were observed at ∼100 nM LPA and 1% FCS (containing up to 50 nM LPA; [3]) (Figure 1A,B). Strikingly, the inhibitory effects were observed only when LPA and FCS were present in the bottom well of the transwell chamber. LPA or serum added to the top well had no detectable effect on cell migration, regardless of the concentration or the direction of the LPA gradient across the filter (Figure 1C). In other words, the inhibitory response to LPA is highly polarized. In keeping with this notion, LPA had no detectable effect on the random motility of B16 cells in low-density culture, as evidenced by time-lapse video microscopy (not shown).

LPA is produced extracellularly from lysophosphatidylcholine (LPC) by autotaxin (ATX). Addition of LPC(18∶1) alone to the lower chamber had no effect, but cell migration was significantly inhibited when LPC was added together with recombinant ATX (Figure 1D). Again, inhibition of migration was only observed when ATX plus LPC were present in the lower chamber.

Even though the basal migration rate of B16 cells is already high, it could be further enhanced by hepatocyte growth factor (HGF). As shown in Figure 1E, LPA reduced the chemotactic activity of HGF by some 60%, but only when both LPA and HGF were present in the lower chamber. This result defines LPA as an anti-chemotactic agonist for B16 cells. Based on these findings, we conclude that LPA inhibits B16 cell migration in a highly polarized fashion, likely reflecting localized LPA receptor signaling in the extending pseudopodia.

**Figure 2:**
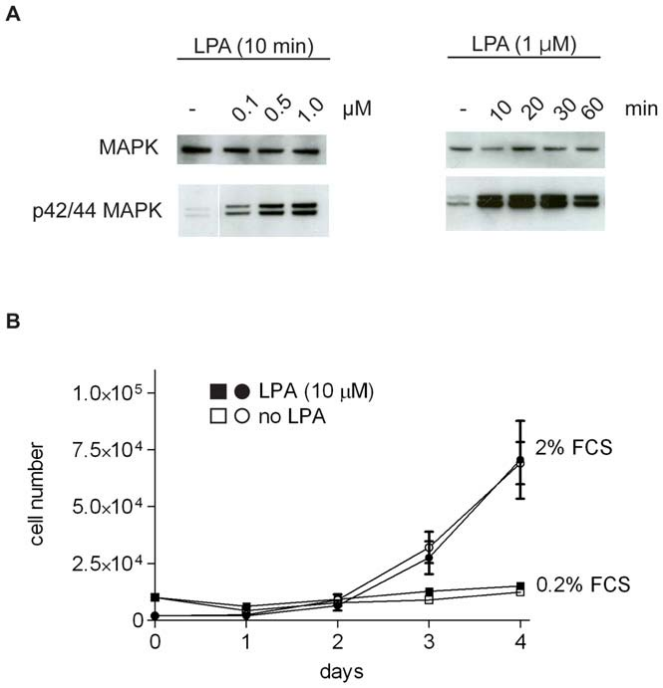
Effect of LPA on ERK1/2 activity and cell proliferation. **A.** LPA-induced activation of ERK1/2 in B16F10 cells. Western blot analysis of total ERK1/2 and phosphorylated ERK1/2 (MAPK), using anti-ERK1/2 and anti-pERK1/2 antibodies. Cells were stimulated with the indicated LPA concentration for the indicated periods of time. The blots are representative of three independent experiments. **B.** Cells were plated at day zero in medium containing 10% FCS. After 16 hrs, the cells were exposed to medium containing 0.2% or 2% FCS, with or without added LPA (10 µM). LPA was refreshed every 24 hrs and cells were counted in triplicate. Values are the means ± SEM (N = 5).

We also tested the effect of LPA on B16 cell proliferation. Melanomas are often characterized by activating mutations in B-Raf, leading to constitutive activation of the mitogenic MAPK/ERK1/2 pathway. B16F10 cells, however, express wild-type B-Raf [23]. Consistent with their normal B-Raf status, B16 cells showed very low basal MAPK/ERK1/2 activity, which was strongly enhanced upon LPA addition (Figure 2A). Yet, LPA (up to 10 µM) did not significantly affect the rate of B16 cell proliferation, either in the presence or absence of limiting concentrations of serum (Figure 2B).

### Dissection of LPA signaling pathways: involvement of cAMP and protein kinase A

**Figure 3:**
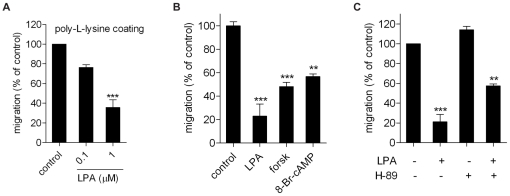
Effect of poly-L-lysine and cAMP reagents on cell migration. **A.** Inhibitory effect of LPA on B16F10 cells plated on poly-L-lysine-coated membranes. **B.** Effects of forskolin (25 µM) and 8-Br-cAMP (100 µM) on transwelll migration of B16F10 cells. **C.** Effect of PKA inhibitor H-89 (30 µM) on cell migration in the presence or absence of LPA (1 µM). Values are the means ± SEM (N = 3; each experiment performed in quadruplo) (**P<0.001; *** P<0.0001).

We next set out to explore the signaling determinants of the chemorepulsive effect of LPA. Optimal transwell migration was observed with cells plated on fibronectin-coated membranes. When B16 cells were plated on poly-L-lysine, which does not interact with integrins, migration was similarly inhibited by LPA (Figure 3A). Thus, the anti-migratory effect of LPA is independent of the nature of the adhesive substrate and, by inference, does not involve specific integrins.

In general, GPCR-mediated cell migration depends on pertussis toxin (PTX)-sensitive G_i_ proteins that couple to activation of Ras- and Rac-mediated signaling pathways. Pretreatment of B16 cells with PTX (200 ng/ml) did not affect the basal cell migration rate nor its inhibition by LPA, which rules out the involvement of G_i_-linked pathways. In addition, we used the Rho-kinase (ROCK) inhibitor Y27632, the protein kinase C inhibitor Ro-31-8220 and two MEK inhibitors (PD598059 and U-0126), which were added to both chambers. However, none of these inhibitors influenced the anti-migratory effect of LPA during a 4 hrs time period (data summarized in Table S1). The lack of effect of Y27632 is somewhat unexpected, since the RhoA-ROCK pathway is known to mediate contractile and anti-migratory events and, furthermore, underlies LPA-induced chemorepulsion of neuronal growth cones; in the latter case, Y27632 can even convert chemorepulsion into chemoattraction [24]. We measured RhoA-GTP levels in B16 cells before and after LPA stimulation, but were unable to detect a significant effect of LPA on RhoA-GTP accumulation in these cells (data not shown).

Given the multifunctional role of cAMP in cytoskeletal regulation and cell migration, we tested the cAMP-raising agents forskolin and 8-Br-cAMP. Both stimuli partly mimicked LPA in inhibiting the basal migration rate of B16 cells (Figure 3B and Table S1), indicating that cAMP is a candidate messenger for mediating LPA-induced chemorepulsion. cAMP acts through activation of protein kinase A (PKA), while at higher levels it exerts additional effects through activation of Epac [25]. Epac is an exchange factor for the Rap GTPase and is activated by direct binding of cAMP. The PKA inhibitor H-89 attenuated the inhibitory responses to LPA and forskolin without affecting the basal migration rate (Figure 3C), whereas the Epac activator 8CPT-2Me-cAMP [26] had no detectable effect on cell migration (Table S1). We confirmed that H-89 inhibits PKA activity by using a FRET-based PKA sensor in forskolin-treated cells as described previously [27].

**Figure 4:**
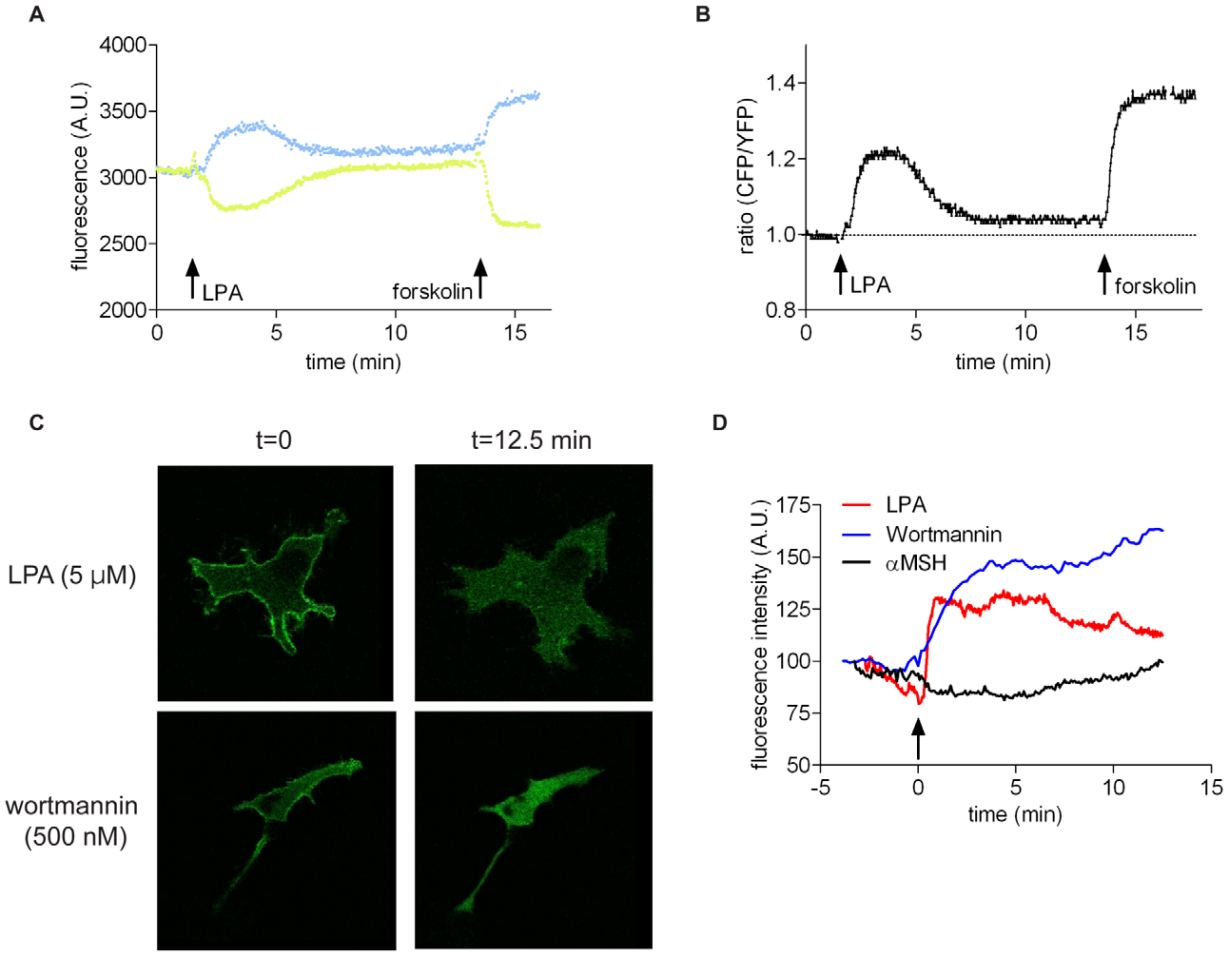
LPA-induced changes in cAMP and PIP3 in B16 cells. **A–B.** Time course of LPA-induced changes in cAMP, monitored by using the FRET-based sensor CFP-Epac-YFP. **A.** Time course of the separate CFP and YFP signals (blue and green, respectively). **B.** An increase in the ratio CFP/YFP reflects an increase in [cAMP] (loss of FRET due to Epac unfolding [27]). LPA, 1 µM; forskolin (10 µM) was used to calibrate the response. **C.** Confocal images of LPA- and wortmannin-induced loss of PIP3 from the plasma membrane, as inferred from the translocation of PH(GRP1)-GFP to the cytosol. See also Video S1. **D.** Agonist-induced increase in cytosolic PH(GRP1)-GFP fluorescence, reflecting loss of PIP3 from the plasma membrane. Dynamic changes in fluorescence intensity (relative to non-stimulated control) were quantified using ImageJ software. LPA, 1 µM; wortmannin, 500 nM; α-MSH, 1 µM.

To monitor changes in cAMP, we used the FRET-based cAMP sensor CFP-Epac-YFP [27]. When expressed in mammalian cells, CFP-Epac-YFP shows a significant FRET signal that rapidly decreases upon a rise in cAMP [27]. In B16 cells expressing this biosensor, LPA induced an initial transient rise in cAMP lasting for about 5 min, followed by a prolonged phase of elevated cAMP levels (Figure 4A,B). From these results we conclude that the cAMP-PKA pathway, rather than the cAMP-Epac-Rap pathway, contributes to LPA-induced chemorepulsion.

### LPA-induced PIP3 depletion from the plasma membrane

In many cases, directed cell migration is critically dependent on the lipid second messenger phosphatidylinositol (3,4,5)-trisphosphate (PIP3), which recruits PH-domain-containing proteins to regulate F-actin remodeling and other key signaling events. Local changes in PIP3 levels can be readily visualized by the redistribution of green fluorescent protein (GFP)-tagged PH domains, such as the biosensor PH(GRP1)-GFP [28]–[30]. Most, if not all, chemoattractants activate phosphoinositide 3-kinase (PI3K) to induce rapid accumulation of PIP3 at the plasma membrane, as detected by the translocation of PIP3-sensitive probes from the cytosol to the plasma membrane [28]–[30]. Using PH-GRP1-GFP as a biosensor, we found that non-stimulated B16 cells show already relatively high basal PIP3 levels at the plasma membrane. LPA triggered a rapid depletion of PIP3 from the plasma membrane as inferred from the translocation of PH(GRP1)-GFP to the cytosol (Figure 4C,D and Video S1). The PI3K inhibitor wortmannin induced a more sustained depletion of PIP3 from the plasma membrane (Figure 4C,D) and inhibited the basal migration of B16 cells (Table S1). We also examined the PIP3-dependent phosphorylation state of Akt (protein kinase B) [31]. Consistent with the PIP3 imaging results, basal pAkt levels were high in non-stimulated B16 cells. LPA induced a modest fall in pAkt levels, while a PI3K inhibitor reduced basal pAkt levels to almost zero (Figure S1).

It thus appears that LPA-induced chemorepulsion of B16 cells is associated with (i) an increase in intracellular cAMP and (ii) PIP3 depletion from the plasma membrane. PIP3 depletion is not secondary to a prior rise in cAMP levels, since the cAMP-raising agonist α-melanocyte-stimulating hormone (α-MSH) (see below) and forskolin failed to trigger PIP3 depletion (Figure 4D and data not shown). Whether LPA-induced PIP3 depletion is due to inhibition of basal PI3K activity or enhanced PIP3 phosphatase activity, or both, remains to be determined.

### Chemorepulsion is mediated by LPA_5_, an alkyl-LPA preferring receptor

**Figure 5:**
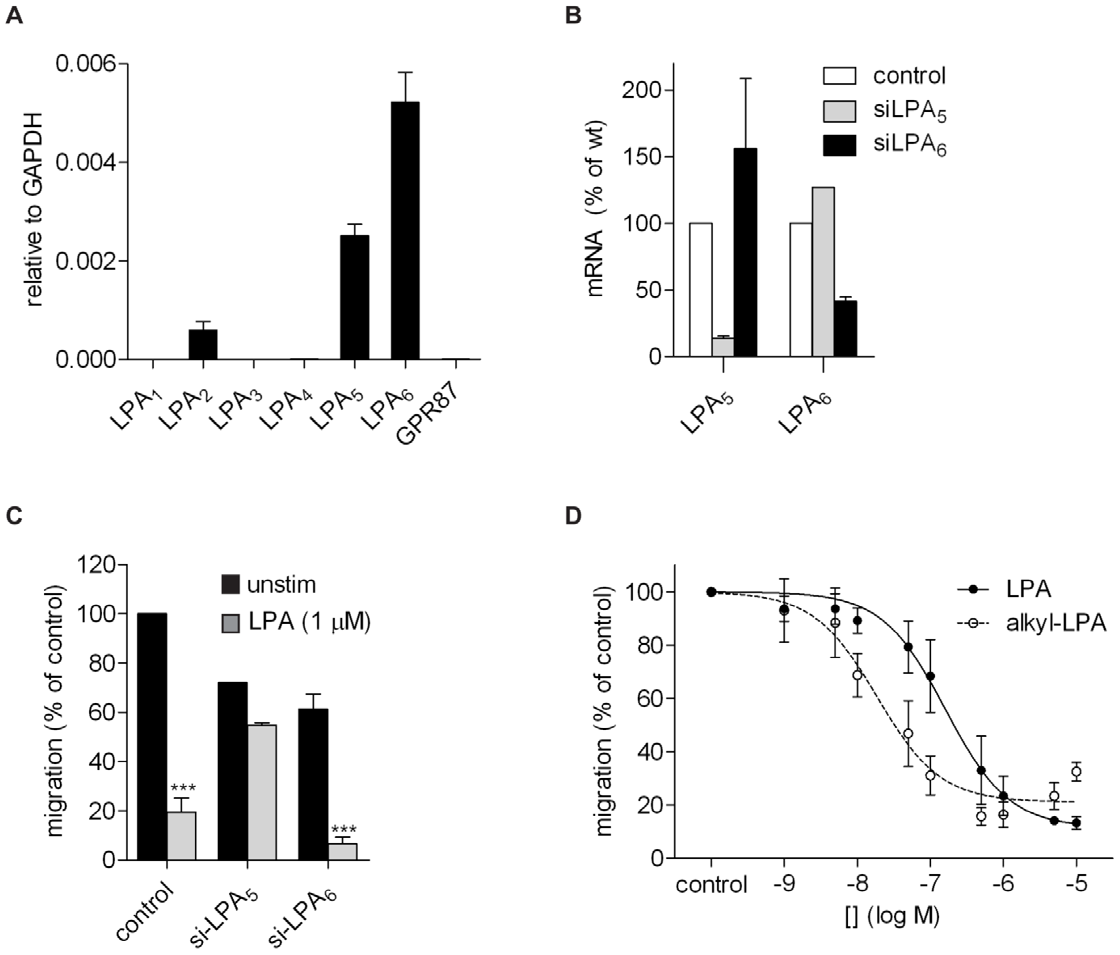
Evidence for LPA_5_ mediating inhibition of cell migration. **A.** LPA receptor expression in B16F10 cells as determined by qPCR. GPR87 is a putative LPA receptor that awaits validation [49]. **B.** Knockdown of LPA_5_ and LPA_6_ expression using specific siRNA pools (see Materials and Methods). Values are relative to GAPDH mRNA levels (**A**) or as percentage (**B**). Values are the means ± SEM (N = 5). **C.** Migration of B16F10 cells transfected with siRNA against LPA_5_ or LPA_6_, or a non-targeting siRNA (control), in the presence or absence of LPA. Relative values are the means (± SEM) of two independent experiments each performed in quadruplo (*** P<0.0001). **D.** Inhibition of cell migration by increasing concentrations of 1-oleoyl-LPA and 1-alkyl-LPA(18∶1). IC_50_ values: alkyl-LPA(18∶1) ∼10 nM; LPA(18∶1) ∼100 nM.

Which LPA receptor(s) mediate(s) the observed chemorepulsive effect? Quantitative PCR analysis revealed that B16 cells co-express LPA_2_, LPA_5_ and LPA_6_, with the relative expression levels of LPA_5_ and LPA_6_ being much higher than that of LPA_2_ (Figure 5A). We used siRNA to knock down LPA_5_ and LPA_6_ and assess the effects on migration. The inhibitory effect of LPA on cell migration was largely abolished after knockdown of LPA_5_, but not after knockdown of LPA_6_ **.** LPA_5_ knockdown did not significantly affect the basal cell migration rate, nor did it alter the expression levels of LPA_2_ and LPA_6_ (Figure 5B,C). This strongly suggests that LPA_5_ mediates the anti-migratory response of B16 cells.

LPA_5_ is unique in that it shows a clear preference for ether-linked LPA over the more common ester-linked LPA molecular species [32], [33]. We therefore compared the efficacy of LPA(18∶1) to that of the corresponding ether-linked analog 1-*O*-alkyl-LPA(18∶1). As shown in Figure 5D, alkyl-LPA(18∶1) was ∼10-fold more potent than acyl-LPA(18∶1) in inhibiting cell migration (IC_50_ ∼10 nM versus 100 nM). This difference in potency cannot be reconciled with Edg-family LPA receptors (LPA_1-3_), which prefer the acyl forms of LPA. This result confirms critical involvement of LPA_5_ in mediating the anti-migratory response to LPA.

**Figure 6:**
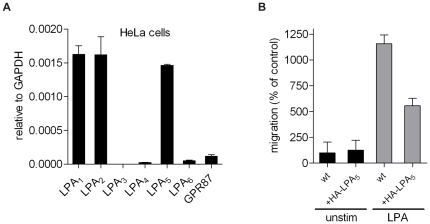
Reduced chemotactic response to LPA in LPA_5_-transfected HeLa cells. **A.** LPA receptor expression in HeLa cells as determined by qPCR. GPR87 is a putative LPA receptor that awaits validation [49]. **B.** Wild-type or HA-LPA_5_-transfected Hela cells were allowed to migrate for 24 hrs in the presence or absence of LPA (1 µM) as indicated. Note reduced chemotaxis in the LPA_5_ transfectants. Values are normalized to migration of unstimulated cells (means ± SEM; N = 4).

To further establish the anti-migratory potential of LPA_5_, we evaluated LPA_5_ in HeLa cells. These cells express LPA_1_, LPA_2_ and LPA_5_ at about equal mRNA levels (Figure 6A), while their basal migration rate is very low (Figure 6B). LPA induced a robust chemotactic response in HeLa cells. In LPA_5_-overexpressing HeLa cells, however, LPA-induced chemotaxis was significantly reduced (Figure 6B). These results support the view that the relative expression pattern of LPA receptors in a given cell type determines migration outcome. That is, the chemorepulsive activity of LPA is observed in those cells where LPA_5_ is the predominant receptor, overriding the chemoattractant activity of LPA_1_ and LPA_2_.

### LPA_5_ mediates an increase in cAMP

Thus far, the signaling properties of LPA_5_/GPR92 have been inferred from heterologous expression studies [34]–[37]. LPA_5_/GPR92-transfected B103 neuroblastoma cells undergo cytoskeletal contraction (Rho kinase-dependent), Ca^2+^ mobilization and an increase in cAMP after LPA addition, whereas control B103 cells do not [35]. The rise in cAMP is not mediated by the canonical Gα_s_ pathway, but most likely involves βγ subunits; there is no evidence for LPA_5_ coupling to G_i_ pathways [35].

**Figure 7:**
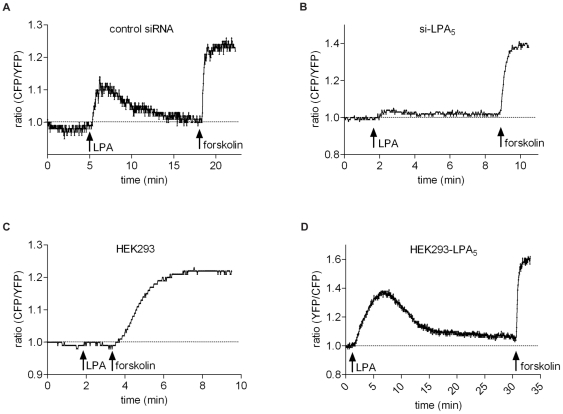
Rise in cAMP mediated by LPA_5_. Cells transfected with CFP-Epac-YFP were stimulated with LPA (1 µM) and forskolin (10 µM) as indicated. The traces depict a cAMP-induced loss of FRET (unfolding of Epac) as an increase in CFP/YFP ratio [27]. **A.** B16 cells transfected with non-targeting siRNA. **B.** B16 cells transfected with siRNA against LPA_5_. **D.** Wild-type HEK293T cells. **E.** HEK293T cells transfected with LPA_5_. All traces are representative of at least three independent experiments.

To assess whether the increase in cAMP in LPA-treated B16 cells (Figure 4B) is mediated by LPA_5_, we monitored cAMP levels in LPA_5_-depleted B16 cells. While the LPA-induced rise in cAMP was not affected in cells treated with non-targeting siRNA, it was absent in LPA_5_ knockdown cells (Figure 7A,B), indicating that LPA_5_ mediates the cAMP signal. We next expressed LPA_5_ in HEK293 cells, as these cells do not normally show a rise in cAMP upon LPA stimulation (Figure 7C). In LPA_5_-transfected HEK293 cells, LPA evoked a significant increase in cAMP with kinetics similar to those observed in B16 cells (Figure 7D).

Taken together, these results demonstrate that LPA_5_ is a cAMP-elevating receptor, both in its native cellular context and in transfected cells, and they reinforce the notion that the anti-migratory activity of LPA_5_ can be attributed, at least in part, to a rise in cAMP with subsequent activation of PKA.

### Comparing LPA to α-MSH and sphingosine 1-phosphate

**Figure 8:**
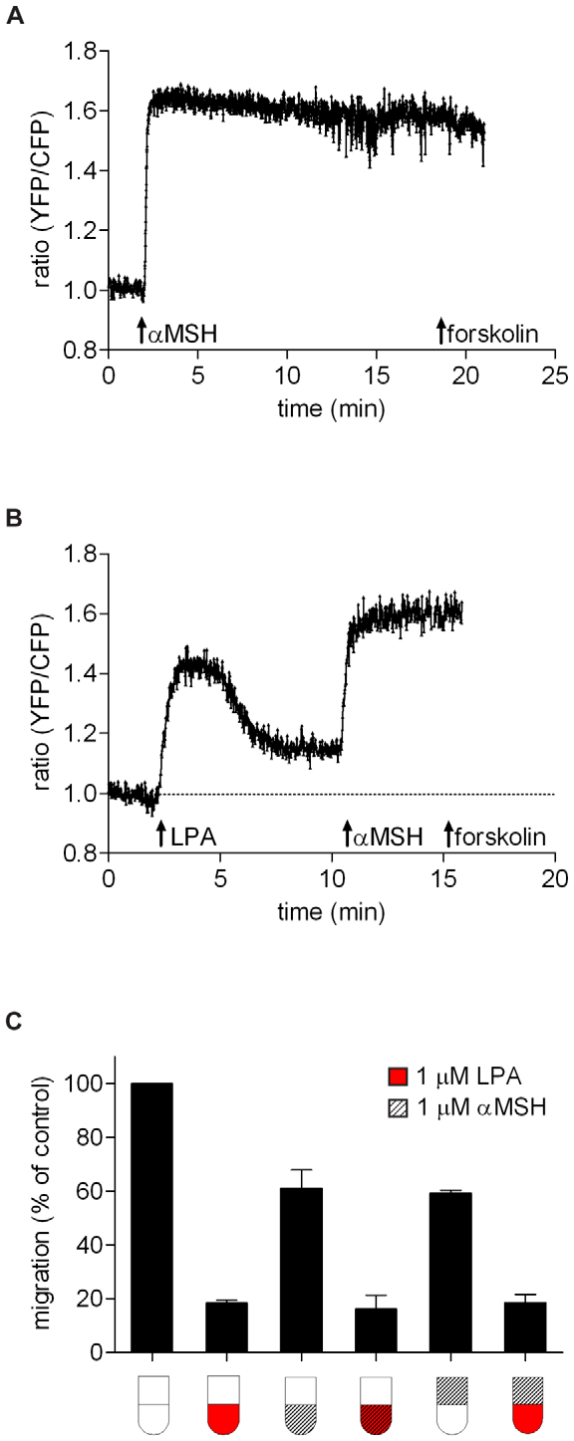
Effects of α-MSH on cAMP and cell migration. **A–B.** B16F10 cells, expressing CFP-Epac-YFP, were stimulated with α-MSH (1 µM), LPA (1 µM) and forskoIin (10 µM) as indicated. **C.** Inhibition of transwell migration by α-MSH, when present in either the upper or the lower chamber as illustrated.

To confirm that cAMP serves as an anti-migratory signal we used α-MSH, an agonist of Gα_s_-coupled melanocortin receptors. It has been reported that α-MSH suppresses the metastatic potential of B16 cells but does not affect their proliferation [38]. α-MSH induced a very rapid and prominent increase in cAMP (Figure 8A,B) and partly inhibited B16 cell migration, regardless of whether α-MSH was present in the lower or upper chamber (Figure 8C); the latter result clearly differs from LPA action. The anti-migratory effect of α-MSH was less pronounced than observed with LPA, while the rise in cAMP is more rapid and more sustained, similar to what is observed with forskolin. The marked difference between the α-MSH- and LPA-induced cAMP signals must reflect a fundamental distinction between the canonical (α_s_) and the noncanonical (βγ) pathway, respectively, by which GPCRs can stimulate cAMP production. Together, these results support the view that cAMP is an anti-migratory signal for B16 cells. However, α-MSH and forskolin appear to act globally, whereas LPA signals in a polarized manner. Furthermore, LPA triggers PIP3 depletion from the plasma membrane, whereas α-MSH does not (Figure 4D).

Inhibition of B16 cell migration is also induced by sphingosine 1-phosphate (S1P), acting through the S1P_2_ receptor, which has been associated with altered Rac/Rho-kinase signaling [39]. Unlike LPA, however, S1P inhibited B16 cell migration when the agonist was present alone in the upper chamber; furthermore, S1P failed to affect basal cAMP levels in B16 cells (Figure S2). This suggests that S1P_2_ and LPA_5_ differ fundamentally in their anti-migratory signaling mechanisms.

### LPA_5_ subcellular localization

Next, we asked whether the polarized response of B16 cells to LPA is attributable to an asymmetrical distribution of LPA_5_. Certain GPCRs show a polarized subcellular localization as exemplified by the P2Y receptor family [40]. In MDCK epithelial cells, a subset of P2Y receptors resides at the basolateral membrane, while other P2Y members are only found at the apical plasma membrane [40]. We examined the distribution of HA-tagged LPA_5_ in MDCK, HeLa and B16 cells. However, none of these cell types showed a clear asymmetric distribution of LPA_5_ (Figure S3). We therefore assume that the polarized response of migrating B16 cells to LPA is a reflection of localized effector pathways in the pseudopodia, rather than of localized LPA_5_ expression.

### Conclusions

How does the cAMP-PKA pathway, with a possible additional role for reduced PIP3 signaling, contribute to the chemorepulsive effect of LPA_5_? PKA can exert both stimulatory and inhibitory effects on cytoskeletal dynamics and cell migration [41], but its action is very complex since PKA acts on numerous target proteins depending on agonist, cell type and subcellular localization of PKA [42]. In epithelial cells, the PKA catalytic subunit (encoded by *PRKCA*) emerged as an anti-migratory gene from RNAi screens [43], [44]. In fibroblasts and carcinoma cells, the cAMP-PKA pathway can inhibit cell migration by interfering with lamellipodia formation [45]. Other studies show that PKA is enriched in protrusive pseudopodia [46], [47], but the specific PKA substrates remain to be identified. Inhibition of cell migration by LPA_5_ most likely occurs through PKA-mediated and PIP3-dependent modulation of components in the Rho/Rac and cytoskeletal assembly pathways. Localized PKA activation, along with PIP3 depletion, would then lead to destabilization and retraction of extending pseudopodia.

Chemoattractants and repellents cooperate during tumor cell migration and metastasis, and hence the outcome of cell migration is dictated by the relative expression levels of individual receptors. While the precise mechanisms underlying LPA_5_-mediated chemorepulsion remain to be determined, the present findings warrant further studies on the expression patterns and clinical outcome of LPA_5_ in melanoma and other cancers. By inhibiting cell migration, LPA_5_-selective agonists might have therapeutic potential for those tumors in which LPA_5_ is a predominant receptor. Caution will be needed, however, since LPA_5_ signaling may also impact on platelet and mast cell functions [32], [33], [48].

## Materials and Methods

### Cell culture and materials

B16F10 melanoma cells were obtained from the American Type Culture Collection (ATCC cat. no. CRL-6475). Cells were cultured in DMEM (Dulbeccos' Modified Eagle Medium) supplemented with 10% fetal calf serum (FCS), L-glutamine and antibiotics. Lysophospholipids were purchased from Avanti Polar Lipids, Inc.; HGF and α-MSH were from Sigma-Aldrich. PH(GRP1)-GFP plasmid was kindly provided by T. Balla (NIH, Bethesda, MD, USA).

### RNA isolation and real-time PCR

Total RNA was extracted using RNeasy Micro Kit (Qiagen). cDNA was synthesized by reverse transcription from 2 µg RNA using oligodT 15 primer and SSII RT enzyme (InVitrogen). RT-qPCR was performed on a 7500 Fast System (Applied Biosystems) as follows: 95°C for 2 min., 95°C for 10 min., 40 cycles at 95°C for 15 sec. followed by 60°C for 1 min. for annealing and extension. The final reaction mixtures (20 µl) consisted of diluted cDNA, 1×SYBR Green Supermix (Applied Biosystems), 200 nM forward primer and 200 nM reverse primer. Reactions were performed in 96-well plates in duplicate. Primer sequences are listed in Table S2.

### RNA interference and transfections

siRNA pools specific for LPA_5_ and LPA_6_ and non-targeting siRNAs were purchased from Dharmacon, Inc. B16F10 cells were transfected with 2 µM siRNA using Dharmafect-2 according to the manufacturer's protocol. Cells were used 3 days after transfection. Transfections of LPA_5_ and CFP-Epac-YFP plasmids were done using Fugene according to the manufacturer's protocol, using 10 µg of DNA on 25 µl of Fugene for a 10-cm dish with 3×10^6^ cells in suspension. Transfection efficiency was determined by side-by-side transfection with GFP and was typically around 75%. PH(GRP1)-GFP was transfected into B16F10 cells using Fugene according to the manufacturer's protocol, using 1 µg of DNA on 3 µl of Fugene6 per well on a 6-well plate. Efficiency was typically around 30%.

### Cell migration assays

Cell migration was measured using 48-well chemotaxis chambers (Neuro Probe, Inc.) equipped with 8 µm-pore polycarbonate membranes, which were coated with either fibronectin or poly-L-lysine (20 µg/ml). Cells (1×10^6^/ml) were added to the upper chamber. Fatty acid-free BSA (1 mg/ml) was used as a lysophospholipid carrier. Migration was allowed for 3 hrs for B16F10 and 24 hrs for HeLa cells at 37°C in humidified air containing 5% CO_2_. Migrated cells were fixed in Diff-Quik Fix and stained using Diff-Quik II. Migration was quantified by color intensity measurements using TINA software.

### Western blotting

Cells were washed in ice-cold PBS (phosphate-buffered saline containing 2 mM Ca^2+^ and Mg^2+^), lysed in RIPA buffer with protease inhibitors and spun down. 15 µl of lysate was loaded onto a 4–12% NuPage® Bis-Tris Gel. Electrophoresis was carried out at 200 V for 40 min in NuPage® MOPS running buffer. Protein was transferred to a nitrocellulose membrane using the iBlot® Dry Blotting System. The membrane was blocked for 1 hour at room-temperature in 50 mg/ml non-fat milk powder in TBST. Incubation with antibodies was done overnight at 4°C. Proteins were visualized using chemiluminescence.

### Monitoring cAMP

Intracellular cAMP levels were monitored in real-time using the FRET-based CFP-Epac-YFP sensor, as described [27]. Transfected cells on coverslips were placed on an inverted NIKON microscope and excited at 425 nm. Emission of CFP and YFP was detected simultaneously through 470±20 and 530±25 nm band-pass filters. Data were digitized and FRET was expressed as the ratio of CFP to YFP signals, the value of which was set to 1.0 at the onset of the experiments. Changes were measured as percentage deviation from this initial value.

### PIP3 imaging

Agonist-induced changes in the levels of phosphatidylinositol (3,4,5)-trisphosphate (PIP3) in the plasma membrane were monitored using a biosensor consisting of the PIP3-specific PH domain of GRP1 (general receptor for phosphoinositides-1) fused to GFP, PH(GRP1)-GFP [28]. PH(GRP1)-GFP-transfected B16F10 cells were grown on glass coverslips and serum starved overnight. Live-cell imaging was done on a Leica TCS SP2 confocal microscope equipped with an Ar 488 laser system. Images were taken using a 63× NA 1.32 objective. Coverslips were mounted in a metal ring system and buffer was applied (140 mM NaCl, 5 mM KCl, 2 mM MgCl_2_, 1 mM CaCl_2_, 23 mM NaHCO_3_, 10 mM HEPES, 10 mM glucose). Cells were selected randomly. Agonists were added while images were collected at appropriate time intervals (2–7.5 sec).

## Supporting Information

Figure S1 **Effect of LPA on pAkt levels in B16F10 cells. A.** Western blot showing the phosphorylation status of Akt (pAkt; Ser473) in non-stimulated and LPA-stimulated B16F10 cells, in the presence or absence of PI3K inhibitor LY294002 (10 µM) as indicated. **B.** Quantification (using TINA software) of the pAkt bands in (**A**) relative to total Akt.
(TIF)

Figure S2 **Effect of sphingosine 1-phosphate (S1P) on cAMP levels in B16F10 cells.** Cells were transfected with the CFP-Epac-YFP cAMP sensor. S1P, 1 µM; forskolin, 10 µM.
(TIF)

Figure S3 **Subcellular localization of LPA_5_ in diverse cell types.** Confocal images of MDCK, HeLa and B16F10 cells tranfected with HA-tagged LPA_5_ and stained for HA (and ZO-1 in MDCK cells) to visualize the localization of LPA_5_. Cells were fixed and permeabilized with methanol at −20°C. Donkey anti-rat A-488 (for LPA_5_) and goat anti-mouse A-594 (for ZO-1) were used as secondary antibodies. Side-views are shown in the right and bottom lanes using Z-stack projections. HA-LPA_5_ in green; ZO-1 in red.
(TIF)

Table S1 **Effect of various reagents on B16F10 cell migration.**
(DOC)

Table S2 **Primer sequences used for qPCR.**
(DOC)

Video S1 **LPA-induced PIP3 depletion from the plasma membrane.** Movie showing LPA-induced loss of PH(GRP1)-GFP from the plasma membrane in LPA-treated B16F10 cells.
(AVI)
