# Supplementary figures and images for: LPA Is a Chemorepellent for B16 Melanoma Cells: Action through the cAMP-Elevating LPA5 Receptor
Source: PLoS One. 2011 Dec 14;6(12):e29260. doi: 10.1371/journal.pone.0029260 (PMC3237609; doi:10.1371/journal.pone.0029260)

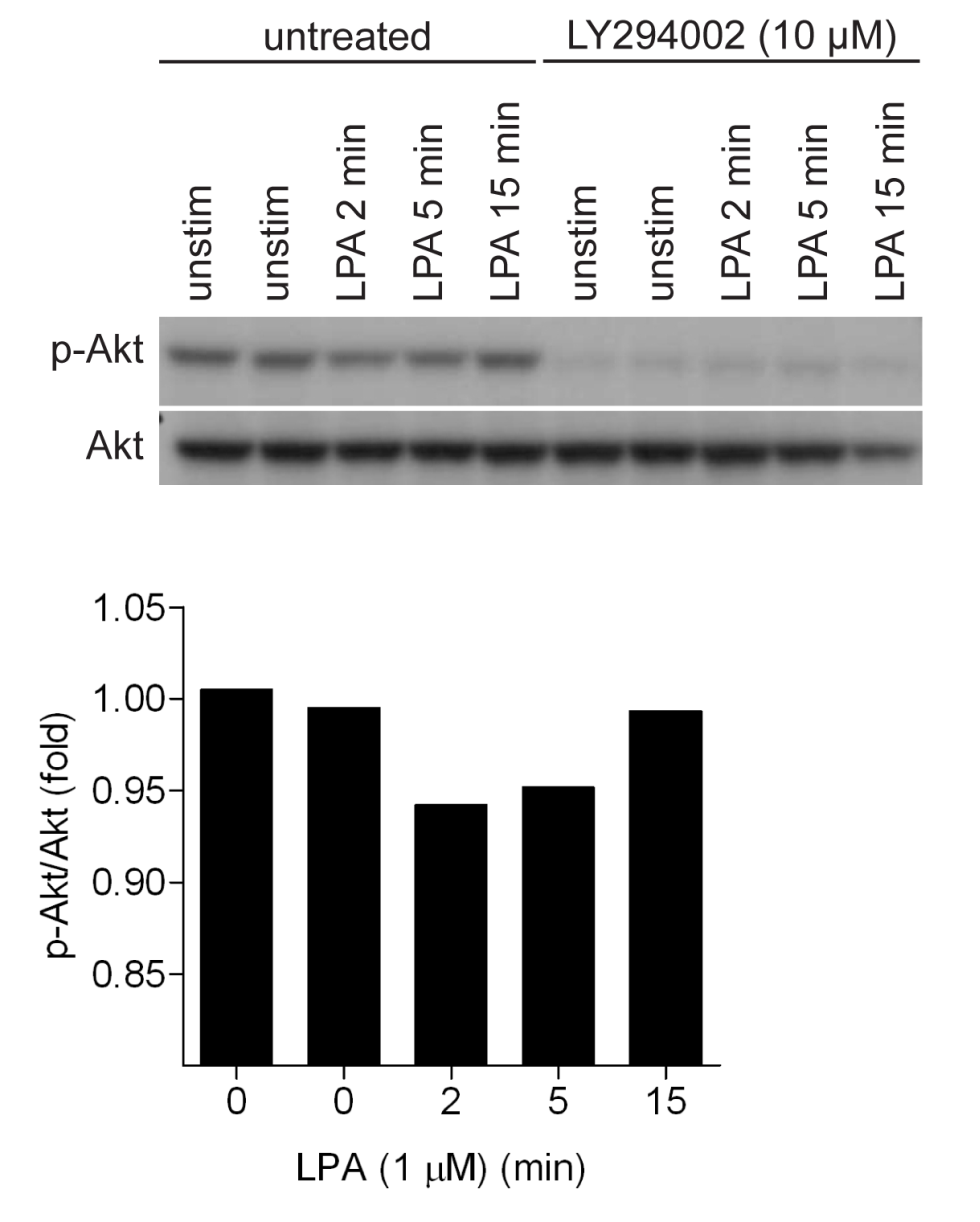

Supplement: Figure S1 — Effect of LPA on pAkt levels in B16F10 cells. A. Western blot showing the phosphorylation status of Akt (pAkt; Ser473) in non-stimulated and LPA-stimulated B16F10 cells, in the presence or absence of PI3K inhibitor LY294002 (10 µM) as indicated. B. Quantification (using TINA software) of the pAkt bands in (A) relative to total Akt. (TIF) [file pone.0029260.s001.tif]

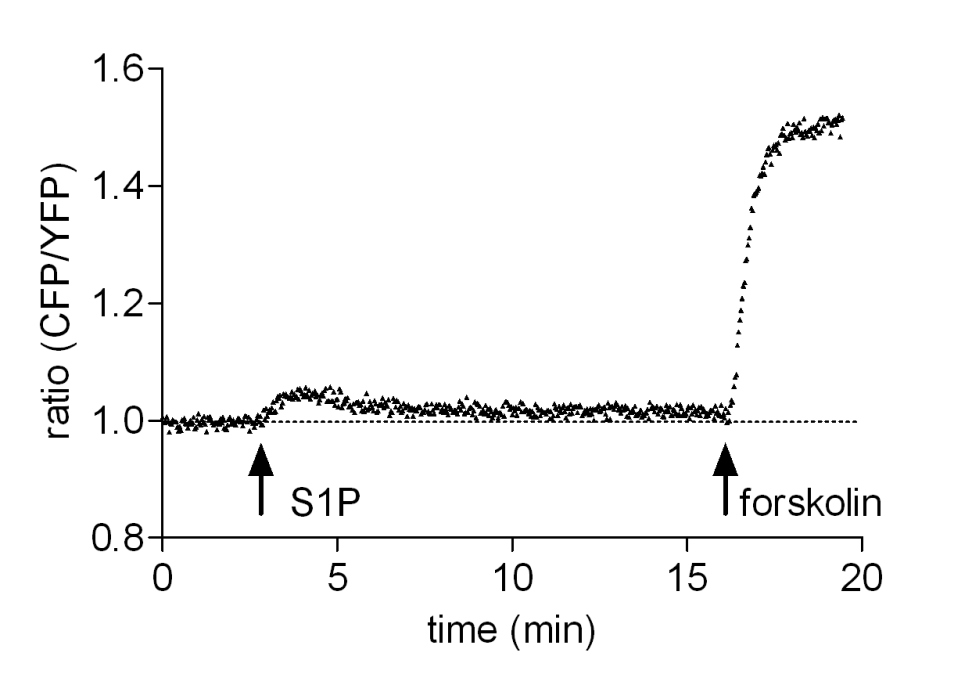

Supplement: Figure S2 — Effect of sphingosine 1-phosphate (S1P) on cAMP levels in B16F10 cells. Cells were transfected with the CFP-Epac-YFP cAMP sensor. S1P, 1 µM; forskolin, 10 µM. (TIF) [file pone.0029260.s002.tif]

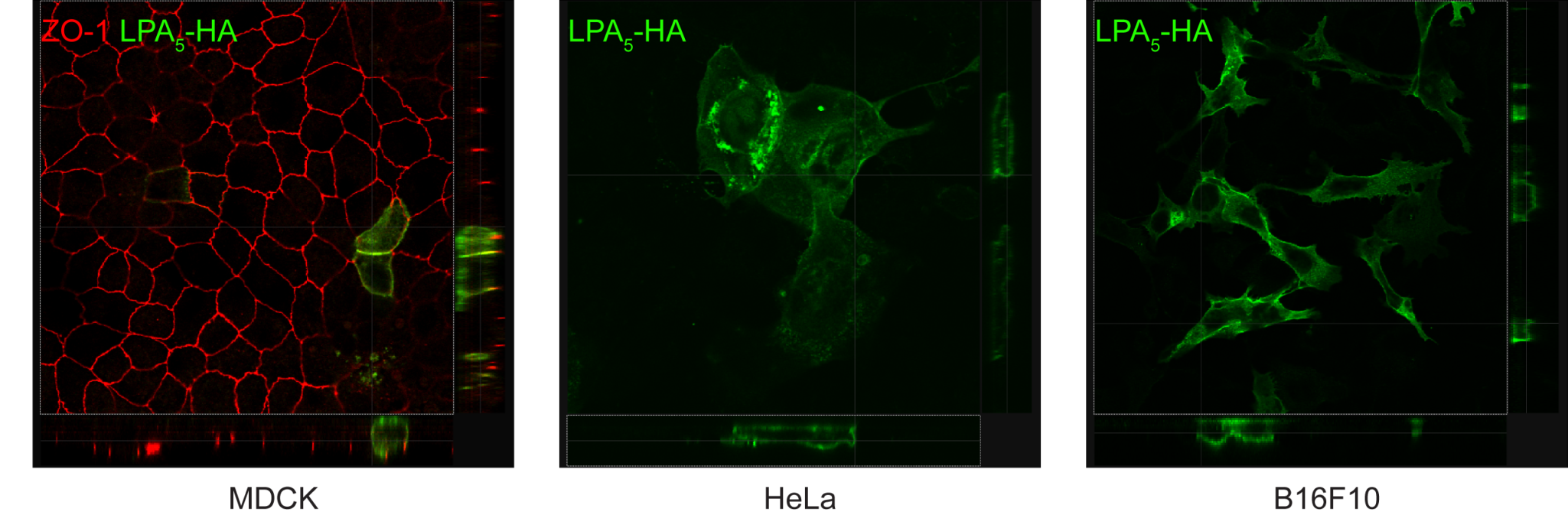

Supplement: Figure S3 — Subcellular localization of LPA5 in diverse cell types. Confocal images of MDCK, HeLa and B16F10 cells tranfected with HA-tagged LPA5 and stained for HA (and ZO-1 in MDCK cells) to visualize the localization of LPA5. Cells were fixed and permeabilized with methanol at −20°C. Donkey anti-rat A-488 (for LPA5) and goat anti-mouse A-594 (for ZO-1) were used as secondary antibodies. Side-views are shown in the right and bottom lanes using Z-stack projections. HA-LPA5 in green; ZO-1 in red. (TIF) [file pone.0029260.s003.tif]
