# Supplementary material for: LPA Is a Chemorepellent for B16 Melanoma Cells: Action through the cAMP-Elevating LPA5 Receptor
Source: PLoS One. 2011 Dec 14;6(12):e29260. doi: 10.1371/journal.pone.0029260 (PMC3237609; doi:10.1371/journal.pone.0029260)
Supplement: Table S1 — Effect of various reagents on B16F10 cell migration. (DOC) [file pone.0029260.s004.doc]

**Table S1. Effect of various reagents on B16F10 cell migration**

| **Agent** | **Concentration** | **Remarks** | **Effect on cell migration** |
| --- | --- | --- | --- |
| LPA(18:1) | 0-10 μM |  | Inhibition up to 90% |
| Serum (FCS) | 0-10% (v/v) |  | Inhibition up to 90% |
| ATX + LPC(18:1) | 10 nM + 5 μM |  | Inhibition, similar to LPA alone |
| LPA + pertussis toxin | 200 ng/ml  (6 hr preincub.) | G_i_ inhibitor | Similar to LPA alone |
| + Ro-31-8220 | 10 μM | PKC inhibitor | idem |
| + Y27632 | 10 μM | Rho-kinase inhibitor | Id  idem |
| + PD598059, U0126 | 10 μM | MEK inhibitors | idem |
| + H-89 | 20 μM | PKA inhibitor | Partial rescue of LPA-induced inhibition |
| + 8CPT-2Me-cAMP | 10 μM | Epac activator | Similar to LPA alone |
| Forskolin | 25 μM | Adenyl cyclase activator | Inhibition (about 50%) |
| 8-Br-cAMP | 100 μM | Cell-permeable cAMP analog | Inhibition (about 50%) |
| α-MSH | 1 μM | cAMP-raising receptor agonist | Inhibition (about 50%) |
| Wortmannin | 100 nM | PI3-kinase inhibitor | Inhibition (about 70%) |
