# Supplementary material for: LPA Is a Chemorepellent for B16 Melanoma Cells: Action through the cAMP-Elevating LPA5 Receptor
Source: PLoS One. 2011 Dec 14;6(12):e29260. doi: 10.1371/journal.pone.0029260 (PMC3237609; doi:10.1371/journal.pone.0029260)
Supplement: Table S2 — Primer sequences used for qPCR. (DOC) [file pone.0029260.s005.doc]

**Table S2. Primer sequences used for qPCR**

|  | Forward | Reverse |
| --- | --- | --- |
| LPA1 human  mouse | AATCGGGATACCATGATGAGTCTT  GAGGAATCGGGACACCATGAT | CCAGGAGTCCAGCAGATGATAAA  ACATCCAGCAATAACAAGAC |
| LPA2 | CGCTCAGCCTGGTCAAGACT  GACCACACTCAGCCTAGTCAAGAC | TTGCAGGACTCACAGCCTAAAC  CTTACAGTCCAGGCCATCCA |
| LPA3 | AGGACACCCATGAAGCTAATGAA  GCTCCCATGAAGCTAATGAAGACA | GCCGTCGAGGAGCAGAAC  AGGCCGTCCAGCAGCAGA |
| LPA4 | CCTAGTCCTCAGTGGCGGTATT  CAGTGCCTCCCTGTTTGTCTTC | CCTTCAAAGCAGGTGGTGGTT GAGAGGGCCAGGTTGGTGAT |
| LPA5 | CGCAATGGCATGTGTGTTC  TCCACGCTGGCTGTATATGG | TCCACGCTGGCTGTATATGG  TCGCGGTCCTGAATACTGTTC |
| LPA6 | AAACTGGTCTGTCAGGAGAAGT  acagtgatgggaggaagtgc | CAGGCAGCAGATTCATTGTCA  ccgctggaaagttctcaaag |
| GPR87 | GCAGGATTTGGACCTTGGTACTTC  tgtgcagaatcccctttacc | TTATCAGCCCAAGGAACAC  gttgcacgcagacaagaaaa |
| GAPDH | GCCAAGGTCATCCATGACAACT | GAGGGGCCATCCACAGTCTT |
